# The reliability of the modified lower extremity functional scale among adults living with HIV on antiretroviral therapy, in Rwanda, Africa

**DOI:** 10.1080/17290376.2014.976249

**Published:** 2014-11-10

**Authors:** D.K. Tumusiime, A. Stewart, F.W.D. Venter, E. Musenge

**Affiliations:** ^a^ PhD, is a Director of Research Centre and Acting Vice Dean of School of Health Sciences, College of Medicine and Health Sciences – University of Rwanda, Rwanda; ^b^ PhD, is an Associate Professor, Department of Physiotherapy, School of Therapeutic Sciences, University of Witwatersrand – Johannesburg, South Africa; ^c^ MD, is a Deputy Executive Director, Witwatersrand Reproductive Health and HIV Institute (WRHI), Department of Medicine, University of the Witwatersrand, Johannesburg, South Africa; ^d^ PhD, is a Biostatistician, Division of Biostatistics and Epidemiology, School of Public Health, Faculty of Health Sciences, University of Witwatersrand, Johannesburg, South Africa

**Keywords:** peripheral neuropathy, HIV, ART, functional activity limitation, reliability of LEFS, Rwanda, Neuropathie périphérique, VIH, TAR, limitation des activitées fonctionnelles, fiabilité de EFMI, Rwanda

## Abstract

Peripheral neuropathy (PN) is common among people living with HIV (PLHIV) on antiretroviral therapy (ART), and affects their daily functional ability and quality of life. Lower extremity functional ability, which is most commonly compromised in patients with PN, has not been clearly evaluated in an African setting, with regard to functional limitations. The lower extremity functional scale (LEFS) was originally developed and validated among elderly people in the USA, where the environment and activities of daily life are very different from those in Rwanda. The purpose of this study was to adapt and establish the reliability of LEFS, among adults living with HIV on ART, in a Rwandan environment. The study translated LEFS from English to Kinyarwanda, the local language spoken in Rwanda, the LEFS was then modified accordingly, and tested for test-retest reliability among 50 adult PLHIV on ART. An average Spearman rank order correlation coefficient, *ρ* ≥ 0.7, was considered optimal for reliability. Prior to the modification of the LEFS and in the initial testing of the translated LEFS, none of the activities was strongly correlated (*ρ* ≥ 0.8); most of the activities (90%, 18/20) were moderately correlated (*ρ* ≥ 0.5) and 10% (2/20) were weakly correlated (*ρ *≤ 0.5). The *ρ* of most of the functional activities improved after modification by an expert group to *ρ* ≥ 0.7, establishing reliability and validity of LEFS among PLHIV on ART with lower extremity functional limitations, in this environment. In conclusion, this study demonstrated the importance of modifying and establishing test – retest reliability of tools derived from developed world contexts to local conditions in developing countries, such as in Rwanda. The modified LEFS in this study can be used in Rwanda by clinicians, specifically at ART clinics to screen and identify people with functional limitations at an early stage of the limitations, for treatment, rehabilitation and/or referral to appropriate health care services.

## Résumé

La Neuropathie périphérique (NP) est fréquente chez les personnes vivant avec le VIH (PVVIH) sous traitement antirétrovirale (TAR), affectant leur capacité fonctionnelle quotidienne ainsi que leur qualité de vie. La capacité fonctionnelle des extremités inferieures qui est la plus compromise chez les patients ayant NP, n'a pas étée clairement évaluée en ce qui concerne les limitations fonctionnelles dans le contexte Africain. L'Echelle Fonctionnelle des Membres Inferieures (EFMI) a été développée et validée sur les personnes âgées aux États-Unis,où l'environnement et les activitées de la vie quotidienne sont très différentes de ceux de l'Afrique. L’ objective generale de cette étude était d'adapter et d’établir la fiabilité de EFMI chez PVVIH sous traitement antirétroviral dans le contexte Africain. Cette étude a traduit EFMI de l’ Anglais en Kinyarwanda , la langue maternelle couramment parlée au Rwanda, et a été modifiée en conséquence, et testée pour une fiabilité test-retest entre 50 PVVIH sous ARV. Un coefficient de corrélation de rang de Spearman moyenne; *ρ* ≥ 0,7 a été considéré comme optimale pour la fiabilité. Avant la modification de EFMI et dans l’étude des tests initiaux de EFMI traduite, aucune des activités a été fortement corrélée (*ρ* ≥ 0,8), la plupart des activités (90%, 18/20) étaient modérément (*ρ* ≥ 0,5) et seulement 10% (2/20) etaient faiblement corrélée (*ρ* ≤ 0,5). Apres la modification par un group d'experts, Le rho de la plupart des activitées fonctionnelles etait amélioré à *ρ* ≥ 0,7, prouvant la fiabilité de EMFI parmi les PVVIH sous ARV avec des limitations des membres inférieurs fonctionnelles dans le contexte Africain . En conclusion, cette étude a démontré l'importance de modifier et d’établir la fiabilité test – retest des outils dérivés de contextes des pays développés, pour les adapter aux conditions locales dans les pays en développement comme en Afrique. EMFI modifiée dans cette étude peut être ainsi utilisée en Afrique sub-saharienne par des cliniciens, en particulier pour TAR cliniques pour dépister et identifier les personnes ayant des limitations fonctionnelles à un stade précoce des limitations, pour le traitement, la réhabilitation et le transfer pour des soins appropriés.

## Introduction

HIV and AIDS continue to result in high levels of mortality and morbidity in sub-Saharan Africa (Joint United Nations Programme on HIV/AIDS [UNAIDS], 2011). They cause various health problems including neurological ones, which are the first manifestations of symptomatic HIV infection in approximately 20–40% of persons (Harrison & Smith 2011; Miura & Kishida 2013). These neurological abnormalities are evident in about 60% of people with advanced HIV disease (McArthur, Brew & Nath 2005). The literature shows that peripheral neuropathy (PN) is the most frequent neurological complication in adults living with HIV (Conradie, Mabiletsa, Sefoka, Mabaso, Louw, Evans, *et al*. 2014; Luciano, Pardo & McArthur 2003; McArthur *et al*. 2005; Sacktor 2002; Simpson, Haidich, Schifitto, Yiannoutsos, Geraci, McArthur, *et al*. 2002; Verma 2001). The introduction of antiretroviral therapy (ART) has resulted in a dramatically reduced mortality rate of people living with HIV (PLHIV) in the countries where this treatment is available (Hogg, Justice, Hayden, Lima, Wasmuth, Harris, *et al*. 2008; Iwuji, Mayanja, Weiss, Atuhumuza, Hughes, Maher, *et al*. 2011; Palella, Delaney, Moorman, Loveless, Fuhrer, Satten, *et al*. 2000). This increased life expectancy and the long-term use of ART have resulted in some chronic complications, which include ART-associated PN becoming more challenging (Arendt & Nolting 2012; Ferrari, Vento, Monaco, Cavallaro, Cainelli, Rizzuto, *et al*. 2006; Morgello, Estanislao, Simpson, Geraci, DiRocco, Gerits, *et al*. 2004). In Rwanda, the prevalence of PN ranges from 40% to 70% among PLHIV on ART (Biraguma & Rhoda 2012; Uwimana & Struthers 2007). PN commonly affects people's daily function and quality of life (QoL) in African populations (Biraguma & Rhoda 2012; Mehta, Ahmed, Kariuki, Said, Omasete, Mendillo, *et al*. 2010).

The LEFS, a tool for evaluating lower limb functional ability, was developed and validated (Binkley, Stratford, Lott & Riddle 1999) in the USA where the environment and activities of daily life are different from those in developing countries such as Rwanda. The activities in developed countries are more urbanised, for instance structured sporting activities, while in developing countries they are more rural, including farming and agricultural activities. To our knowledge, this tool has not been validated in any African country. The purpose of this study was to re-establish the reliability of the modified LEFS so as to assess functional ability of the lower extremity among adults living with HIV and on ART in Rwanda. The specific objectives measured in this study were to translate the LEFS into Kinyarwanda, to test the translated LEFS for clarity, to modify and rectify unclear items found in the LEFS for specific Rwandan cultural activities of daily living (ADL) and thereafter re-establish the intra- and inter-assessor reliability of the modified LEFS.

## Methods

The LEFS assesses the subjective functional activity performance of daily living, in the lower extremities. It was developed and validated for a variety of lower extremity conditions based on the WHO model of impairment, disability and handicap (Binkley *et al*. 1999) particularly for the elderly. The LEFS is expected to measure even small effects of impaired activity performance experienced by participants with lower extremity musculoskeletal dysfunction accurately (Cacchio, De Blasis, Necozione, Rosa, Riddle, di Orio, *et al*. 2010; Yeung, Wessel, Stratford & Macdermid 2009). The scale assesses the level of difficulty in performing a variety of ADLs. Each activity on the scale is scored by the participant as 0 = ‘Extreme difficulty or unable to perform activity’, 1 = ‘quite a bit of difficulty’, 2 = ‘Moderate difficulty’, 3 = ‘A little bit of difficulty’ and 4 = ‘no difficulty’. The scale scores vary from 0 (none) to 80 (normal) (Binkley *et al*. 1999).

## LEFS translation into Kinyarwanda

The scale was translated from English to Kinyarwanda, by two independent professional language translators from the Language Centre at the College of Medicine and Health Sciences, University of Rwanda (CMHS-UR). Susequently, two independent professional translators translated the scale back to English, to ensure content validity. The translation was assessed by a consensus panel of two physiotherapists and two medical doctors working at the Treatment, Research and AIDS Centre in Rwanda, together with all four translators and the first author of this study. Changes and modifications (indicated in the appendices; Table A2) were made for some scientific terms and functional activities in the scale. The modifications in the activity performance were based on the ADLs that are culturally applicable; an example being a question that asked about having difficulty ‘getting in and out of a car’. Most people in Rwanda travel in public taxis/buses (for those who manage to travel in vehicles). When participants were asked about having diificulty getting in and out of a car, some mentioned, ‘I have never moved with a car’ or ‘I seldom move with a car’ So, the item/activity ‘getting in and out of a car’ was modified as having any difficulty of ‘getting in and out of a car/public taxi/bus’.

## Intra-assessor reliability prior to modification of LEFS

A pilot study was carried out to assess for intra- and inter-assessor reliability of the LEFS. Stage 1 of the study aimed at testing the LEFS for intra-assessor reliability. The translated and content-modified LEFS was administered to a sample of 50 adults (18–60 years old) PLHIV on ART, both males and females, who were systematically selected from all the PLHIV on ART registered at an outpatient ART clinic at the Biryogo Health Centre, commonly known as ‘Kwa Nyiranuma’ in Kigali city. The health centre attends to more than 50 PLHIV on ART on each of the five working days of the week. Ten participants were systematically selected from each list of the first 50 PLHIV attending the centre per day. The selection took from the 5th person and systematically with an interval of 5 up to the 50th person on the list. The sample of 50 participants was obtained in one week. This sample size was the optimal number for feasible pilot study data that are scheduled for only one week. Participants with known deformities and injuries of the lower extremities were excluded from the study. The first author administered the translated LEFS to these selected participants. Two assessments were conducted for each participant, with a week's interval between the two assessments. Participants who could read and write were given the scale to complete with the assessor available for clarification of the scale. Participants who did not know how to read or write had the first author administer the scale by reading each question/item to the participant and recording the responses appropriately. These interviews took place in a private room. Prior to the start of assessing the participants in the pilot study, the assessor had practiced scoring of the scale on five adult PLHIV on ART at the same clinic who were not included in the study. This was done to familiarise the administration and scoring techniques of the scale so as to minimise errors.

## Modification of the functional activities in the LEFS

Following the analysis of the intra-assessor correlation between the first and second assessments in stage one, all activities were classified as strong (*ρ* ≥ 0.8), moderate (*ρ* < 0.8 and ≥ 0.5) and weak (*ρ* < 0.5). In addition, during stage one, some activities in the LEFS were unclear to the Rwandan participants and needed precise examples, forming the basis for the subsequent modifications. All such activities were modified and made clearer with specific examples, without changing the concepts and context of the original LEFS. The modification was done in consultation with a team of three health professional experts, two physiotherapists and a medical doctor, who were experienced in rehabilitation services, and two participants. The purpose of the team consultation was to establish appropriate activities that are commonly and culturally undertaken by people living in Rwanda and similar to the activities that define the LEFS. The activities and their common examples were identified.

## Intra- and inter-assessor reliability after modification of LEFS

The modified LEFS was then assessed after modifying unclear, moderate and weakly correlated activities in the scale. The intra- and inter-assessor reliability was undertaken by three assessors; the first author and two other assessors who were qualified physiotherapists with master's degrees and who were selected by the first author. A sample of 12 participants was randomly selected from both female and male adult PLHIV attending ART clinic at the Kanombe Military Hospital in Kigali, by using random numbers that corresponded to the registration numbering list of the participants at the clinic on one day. Two assessments, one week apart, were carried out to test the intra- and inter-assessor reliability after the above modifications. A two-hour training session was conducted for the two assessors to familiarise them with using the scale. The three assessors administered the scale piloted and modified in stage one. Each assessor carried out the assessment of each participant independently, and was blinded to the other assessors' assessment outcomes and participants' scores.

An ethical clearance certificate (protocol number M080812) for this study was obtained from the Human Research Ethics Committee at the University of the Witwatersrand and the research protocol was approved by the Faculty of Health Sciences at the University. As the research data were collected in Rwanda, national clearance was also obtained from the Institutional Review Board at the College of Medicine and Health Science, University of Rwanda, and scientific approval by the National Commission for control of HIV/AIDS, in Rwanda. Authorisation letters were obtained from the Biryogo Medical Centre and Kanombe Military Hospital where the study was conducted. A letter containing information describing the details of the study was given to the participants to invite them to participate, before they were recruited into the study. Participants, who agreed to participate and gave permission for use of their medical records, signed a consent form. Confidentiality and anonymity were ensured for all participants.

The statistical analysis was done using STATA (version 11, Stata Corp, College Station, TX, USA). The variables (activities) in the LEFS were categorical and ordinal in nature. Spearman's rank correlation coefficient was used to measure statistical independence between the same functional activities at the two assessments done at two intervals for the same participants. The activity correlation coefficients were classified according to the levels of strength, as strongly (*ρ* ≥ 0.7), moderately (*ρ* < 7 ≥ 0.5) and weakly (*ρ* < 0.5) correlated activities. All activities with moderate and weak correlation coefficients (*ρ*), according to the classification, in stage one were considered for modification (Table A2).

## Results

Out of the sample of PLHIV (*n* = 50) who underwent the first assessment in stage one, 42 (84%) returned for the second assessment, and these were included in the test-retest analysis for intra-assessor reliability. None of the activities were strongly correlated (*ρ* *≥* 0.7), most of the activities (90%, 18/20) were moderately (*ρ* ****< 7 and  ≥ 0.5) correlated and 10% (2/20) were weakly correlated (*ρ* ≤ 0.5) (Table A1). Activities that were moderately correlated were ‘doing daily home activity’ (*ρ* = 0.57), ‘having recreational/leisure activities’ (*ρ* = 0.63), ‘walking between rooms’ (*ρ* = 0.54), ‘squatting’ (*ρ* = 0.61), ‘lifting small object’ (*ρ* = 0.53), ‘doing light activity at home’ (*ρ* = 0.61), ‘doing heavy activity at home’ (*ρ* = 0.52), ‘getting into & out of car’ (*ρ* = 0.53), ‘walking a km’ (*ρ* = 0.66) and ‘sitting for an hour’ (*ρ* = 0.63), while the weakly correlated activities were ‘putting on shoes & socks’ (*ρ* = 0.44) and ‘walking across from one building to another’ (*ρ* = 0.47). The activities with moderate or weakly correlated coefficients were either unclear or not commonly used in Rwanda, as assessed by the expert committee. Such activities were further modified with specific examples of related activities (column 2) which are culturally appropriate and commonly used by people in Rwanda, to make them clearer. As rated by the three assessors the results (Table A3) indicate that almost all the weak and moderate correlated activities were improved to *ρ* ≥ 0.7, with only one ‘Making sharp turns while walking/running very fast’ *ρ* = 0.62 (*p* = 0.06) remaining moderately correlated.

## Discussion

This study represents the first reliability test of the LEFS in patients on ART, from English (Binkley *et al*. 1999) into Kinyarwanda and adapted for an appropriate cultural context. HIV-related disability has been associated with decreased physical functioning and has numerous impacts on ADLs (Cacchio *et al*. 2010; Cade, Peralta & Keyser 2004). The identification of functional activities of the lower extremity is crucial for rehabilitation of patients with chronic illness such as those living with HIV and on ART (Dudgeon, Phillips, Bopp & Hand 2004; O'Brien, Nixon, Tynan & Glazier 2010). This study tested the LEFS to assess the functional activities of lower extremity for rehabilitation purposes in Rwanda. The tested scale can likely be adapted for similar purposes in Africa and other developing countries. The LEFS has very high correlation coefficient (*ρ* = 0.94) in the developed world. It was developed and validated for the purpose of identification and evaluation of lower extremity functional activity among the elderly (Binkley *et al*. 1999). Studies suggest that there might be important differences in health-related activities between high-income and middle/low-income countries (Karlsson, Nilsson, Lyttkens & Leeson 2010). Scales may not identify the activities among the population in a developing environment (Ebrahim & Davey 2001). According to Ebrahim and Davey (2001) research findings from developed settings are not necessarily appropriate to other contexts; thus, local knowledge is important. Our study confirms this, with most of the activities in the original LEFS being only moderate correlations and a few weak. This was probably attributable to the fact that some of the activities in the LEFS were not familiar to most of the population living in Rwanda. In addition, these differences might be reflective of linguistic specificities and cultural differences, but they may also result from methodological disparities such as differences in the clinical characteristics of patients, as reported by Perez, Galvez, Huelbes, Insausti, Bouhassira, Diaz, *et al*. (2007) in their study which tested the reliability of the Spanish DN4 version from the original French version. It is important that outcome measures used in an environment that is different from the one in which they were originally developed and validated are modified and re-tested. The reliability of the adapted Kinyarwanda version of the LEFS-Modified tool was strong. This implies that the tool can be used by clinicians working at ART clinics, to identify PLHIV with functional limitations at an early stage for appropriate management. Rehabilitation professionals can also use this tool to evaluate progress during rehabilitation. This may improve the quality of care of PLHIV.

## Conclusion

Our modified, translated LEFS performed well, with very few remaining moderate and no weak correlations of functional activities in the local environment. Modifications to take into account local conditions are critical for the evaluation of tools that have been validated in developed world contexts. This study modified and re-tested the reliability of the LEFS tool derived from a developed world context, to local conditions in a developing African country. This implies that the modified LEFS can be well used by clinicians, specifically at the ART clinics in Rwanda and possibly other sub-Saharan African countries to screen and identify people with functional limitations at an early stage, for treatment, rehabilitation and or referral to appropriate health-care services, with the aim of improving the QoL of PLHIV.

## Appendix

**Table A1.** The intra-assessor reliability by the first author (assessor 1) in pilot stage 1 (*n* = 42).

Activities	*ρ*	*p*-Value
Doing daily home activity	0.57^a^	<0.01
Recreational activity	0.63^a^	<0.01
Bath limitation	0.69^a^	<0.01
Walking between rooms	0.54^a^	<0.01
Putting on shoes and socks	0.44^b^	0.02
Squatting	0.61^a^	<0.01
Lifting small object	0.53^a^	<0.01
Doing light activity at home	0.61^a^	<0.01
Doing heavy activity at home	0.52^a^	0.01
Getting into and out of car	0.53^a^	<0.01
Walking across from one building to another	0.47^b^	<0.01
Walking a km	0.66^a^	<0.01
Climbing stairs (10 stairs)	0.63^a^	<0.01
Standing for 1 hour	0.63^a^	<0.01
Sitting for 1 hour	0.65^a^	<0.01
Running on even ground	0.62^a^	<0.01
Running on uneven ground	0.60^a^	<0.01
Making sharp turn while running	0.62^a^	<0.01
Hopping	0.65^a^	<0.01
Roll over in bed	0.68^a^	<0.01

^a^Moderate correlation coefficient.

^b^Weak correlation coefficient.

**Table A2.** Modification of weak and moderate correlated activities, with pilot stage 1 coefficient results.

Activities	*ρ*	*p*-Value	Comments and suggested modifications
Moderately correlated activities and their modifications			
Doing daily usual activity	0.57	<0.02	Specific examples related to activities the person does daily, such as those done at work/employment, going to/coming from work, etc.
Recreational/leisure activity	0.63	<0.01	Most people are not involved in traditionally defined recreational/leisure activities; related activities were given as an example, such as attending weddings and visiting friends, going to church, etc.
Bath limitation	0.79	<0.01	Examples included taking shower or bath
Squatting	0.61	<0.01	A specific example is squatting on pit latrine
Doing light activity around the home	0.61	<0.01	Examples such as preparing a meal, cleaning a house, making a bed
Walking a km	0.66	<0.01	Examples such as going to market/shops, church, or other social activities
Climbing stairs (10 stairs)	0.63	0.00	As an alternative to climbing stairs, additional examples included walking on a relatively steep irregular ground
Standing for 1 hour	0.63	0.00	Examples included standing doing some work, for example, digging, standing on long waiting service lines, shopping
Sitting for 1 hour	0.65	0.00	Sitting for 1 hour, like when in church, public bus/taxi or meetings
Walking between rooms	0.54	<0.01	Specific examples, such ‘walking from bed room to toilet’, bath room, etc.
Lifting an object like a bag of groceries	0.53	<0.01	Examples such as lifting a small container full of water (5 litre gerrican), basket of potatoes, etc.
Doing heavy activity at home	0.52	<0.01	Examples of heavy activities include digging, lifting a heavy bag of potatoes, 20 litre gerrican of water, shifting big items, etc.
Getting into and out of car	0.53	<0.01	Inclusive of public taxi/bus which is common mode of transport for majority
Running on even ground	0.62	0.00	Fast walking on even ground
Running on uneven ground	0.60	0.00	Fast walking on uneven ground
Making sharp turn while running	0.62	0.00	Making sharp turns while walking fast at your pace
Hopping	0.65	0.00	Standing up very fast from squatting as needed
Rolling over in bed	0.68	0.00	Turning in bed
Weakly correlated variables and their modifications			
Putting on shoes and socks	0.44	0.02	Some people do not put on socks or even closed shoes. Question rephrased as ‘ … problems with putting on any kind of shoes, including sandals, etc.’
Walking two blocks	0.47	0.01	A specific distance of 100 m, or walking from his/her home to neighbour's, a distance of not more than 200 m away

**Table A3.** Activities in the original LEFS versus the activities in the modified LEFS.

Original LEFS activities	Modified LEFS activities
Any of your usual work, housework or school activities	Any of your usual work, (e.g. work that earns you income or any other work you do) housework or school activities
Your usual hobbies, recreational or sporting activities	Your usual hobbies, recreational or sporting activities, for example, attending weddings, church or visiting friends
Getting into or out of the bath	Getting into or out of the bath/taking bath
Walking between rooms	Walking between rooms (such as walking from your room to toilet, bathroom, kitchen, etc.)
Putting on your shoes or socks	Putting on any kind shoes or socks you want, including slippers or open shoes, if applicable
Squatting	Squatting (e.g. squatting on pit latrine/doing any squatting activity)
Lifting an object like a bag of groceries from the floor	Lifting an object like a bag of groceries or a small container such as a 5-litre container full of water, basket of potatoes, etc. from floor
Performing light activities around your home	Performing light activities around your home(such as preparing a meal, cleaning a house, making a bed or any other light activity at home)
Performing heavy activities around your home	Performing heavy activities around your home (digging, lifting a heavy bag of potatoes, 20-litre gerrican of water, shifting big items, etc.
Getting into or out of a car	Getting into or out of a car/public taxi/bus
Walking two blocks	Walking across from your home to a neighbour's or walk about 100 m across
Walking a mile	Walking a km, such as going to market, church or any other place
Going up or down 10 stairs (about 1 flight of stairs)	Going up or down 10 stairs (about 1 flight of stairs) or walking up steep and irregular ground
Standing for 1 hour	Examples include standing doing some work, for example, digging, standing in long waiting service lines, shopping, etc.
Sitting for 1 hour	Sitting for 1 hour, like when in church, public bus/taxi or meetings
Running on even ground	Fast walking on even ground
Running on uneven ground	Fast walking/running on uneven ground
Making sharp turns while running fast	Making sharp turns while walking/running very fast
Hopping	Standing up fast from squatting as needed
Rolling over in bed	Turning in bed

**Table A4.** Intra-assessor reliability with Spearman's rank correlation coefficient (*ρ*) and *p*-values, obtained for each assessor for each functional activity in LEFS-Modified, in both assessment 1 and assessment 2.

	Assessor 1		Assessor 2		Assessor 3	
Functional activities	*ρ*	*p*-Value	*ρ*	*p*-Value	*ρ*	*p*-Value
Any of your usual work, (e.g. work that earns you income or any other work you do) housework or school activities	0.9	<0.01	0.75	0.03	0.91	0.02
Your usual hobbies, recreational or sporting activities, for example, attending weddings, church or visiting friends	0.7	0.02	0.82	<0.01	0.83	0.05
Getting into or out of the bath/taking bath	1.00	<0.01	0.99	<0.01	0.73	0.03
Walking between rooms (such as walking from your room to toilet, bathroom, kitchen, etc.)	1.00	<0.01	0.97	<0.01	0.7	0.05
Putting on any kind of shoes or socks, including slippers or open shoes, if applicable	0.9	<0.01	0.80	<0.01	1.0	0.01
Squatting (e.g. squatting on pit latrine/doing any squatting activity)	0.7	0.04	0.76	0.02	0.76	0.04
Lifting an object, like a bag of groceries or a small container such as a 5-litre container full of water, basket of potatoes, etc., from floor	0.72	0.03	0.81	0.03	0.86	0.03
Performing light activities around your home (such as preparing a meal, cleaning a house, making a bed or any other light activity at home)	0.8	0.04	0.72	<0.01	0.7	0.05
Performing heavy activities around your home (digging, lifting a heavy bag of potatoes, 20-litre gerrican of water, shifting big items, etc.)	0.7	0.03	0.77	0.03	0.95	0.02
Getting into or out of a car/taxi	0.8	0.02	0.75	0.04	0.84	0.03
Walking across from your home to a neighbour's or walk about 100 m across	0.7	0.05	0.88	0.02	0.75	<0.01
Walking a km, such as going to the market, church or any other place	0.9	<0.01	0.84	0.02	0.85	<0.01
Going up or down 10 stairs (about 1 flight of stairs) or walking up steep and irregular ground	0.78	0.05	0.78	<0.01	0.73	0.04
Standing for 1 hour	0.9	<0.01	0.83	0.03	0.84	0.03
Sitting for 1 hour, like when in church, taxi or meetings	0.8	0.02	0.70	0.04	0.7	0.03
Fast walking on even ground	0.7	0.04	0.71	0.03	1.0	<0.01
Fast walking/running on uneven ground	0.76	0.02	0.90	<0.01	0.78	0.03
Making sharp turns while walking/running very fast	0.75	<0.01	0.82	<0.01	0.62^a^	0.05
Standing up fast from squatting	1.00	<0.01	0.90	<0.01	1.00	<0.01
Turning in bed	0.8	<0.01	0.88	0.02	0.8	0.02

^a^Moderately correlated.

**Table A5.** Inter-assessor reliability with Spearman's rank correlation coefficient (*ρ*) and *p*-values, obtained for a pair of assessors for each functional activity in LEFS-Modified.

Functional activities	Assessor 1 and Assessor 2		Assessor 1 and Assessor 3		Assessor 2 and Assessor 3	
	*ρ*	*p*-Value	*ρ*	*p*-Value	*ρ*	*p*-Value
Any of your usual work, (e.g. work that earns you income or any other work you do) housework or school activities	0.8	0.01	0.8	0.01	0.8	0.02
Your usual hobbies, recreational or sporting activities, for example, attending weddings, church or visiting friends	0.75	0.01	0.68^a^	0.04	0.9	0.01
Getting into or out of the bath/taking bath	1.0	<0.01	1.00	<0.01	1.00	<0.01
Walking between rooms (such as walking from your room to toilet, bathroom, kitchen, etc.)	1.0	<0.01	1.00	<0.01	1.00	<0.01
Putting on any kind shoes or socks, including slippers or open shoes, if applicable	0.7	0.01	0.69	0.03	0.7	0.02
Squatting (e.g. squatting on pit latrine/doing any squatting activity)	0.74	0.02	0.72	0.04	0.8	0.01
Lifting an object, like a bag of groceries or a small container like a 5-litre container full of water, basket of potatoes, etc., from floor	0.82	0.01	0.67	0.05	1.00	<0.01
Performing light activities around your home (such as preparing a meal, cleaning a house, making a bed or any other light activity at home)	0.72	0.02	0.71	0.03	1.00	<0.01
Performing heavy activities around your home (digging, lifting a heavy bag of potatoes, 20-litre gerrican of water, shifting big items, etc.	0.71	0.03	0.9	0.01	1.0	<0.01
Getting into or out of a car/taxi	0.8	0.01	0.7	0.01	0.7	0.05
Walking across from your home to a neighbour's or walk about 100 m across	0.7	0.01	1.00	<0.01	1.00	<0.01
Walking a km, such as going to market, church or any other place	0.7	0.02	0.83	0.02	1.0	<0.01
Going up or down 10 stairs (about 1 flight of stairs) or walking up steep and irregular ground	0.8	0.01	0.7	0.03	1.0	0.01
Standing for 1 hour	0.86	0.01	0.9	0.05	0.71	0.03
Sitting for 1 hour, like when in church, taxi or meetings	0.73	0.02	0.81	0.01	1.0	0.01
Fast walking on even ground	0.86	0.01	0.7	0.03	1.0	<0.01
Fast walking/running on uneven ground	0.73	0.03	0.74	0.03	1.0	<0.01
Making sharp turns while walking/running very fast	0.9	<0.01	0.85	0.02	1.0	<0.01
Standing up fast from squatting	0.9	0.01	1.0	0.01	1.0	0.05
Turning in bed	0.81	0.01	0.82	0.02	1.00	<0.01

^a^Moderately correlate.

**Table A6.** LEFS-Modified.We are interested in knowing whether you are having any difficulty at all with the activities listed below because of your lower limb problem (s). **Please provide an answer for each activity.** Today, do you or would you have any difficulty with:(Circle one number on each line that corresponds to your appropriate answer)

Activity	Unable to perform activity	Quite a bit of difficulty	Moderate difficulty	A little a bit difficulty	No difficulty
1. Any of your usual work, (e.g. work that earns you income or any other work you do) housework or school activities	0	1	2	3	4
2. Your usual hobbies, recreational or sporting activities, e.g. attending weddings, church or visiting friends	0	1	2	3	4
3. Getting into or out of the bath/taking bath	0	1	2	3	4
4. Walking between rooms (such as walking from your room to toilet, bathroom, kitchen, etc.)	0	1	2	3	4
5. Putting on any kind shoes or socks, including slippers or open shoes, if applicable	0	1	2	3	4
6. Squatting (e.g. squatting on pit latrine/doing any squatting activity)	0	1	2	3	4
7. Lifting an object, like a bag of groceries or a small container like a 5-litre container full of water, basket of potatoes, etc., from floor	0	1	2	3	4
8. Performing light activities around your home (such as preparing a meal, cleaning a house, making a bed or any other light activity at home)	0	1	2	3	4
9. Performing heavy activities around your home (digging, lifting a heavy bag of potatoes, 20-litre gerrican of water, shifting big items, etc.	0	1	2	3	4
10. Getting into or out of a car/taxi	0	1	2	3	4
11. Walking across from your home to a neighbour's or walk about 100 m across	0	1	2	3	4
12. Walking a km, such as going to market, church or any other place	0	1	2	3	4
13. Going up or down 10 stairs (about 1 flight of stairs) or walking up a steep and irregular ground	0	1	2	3	4
14. Standing for 1 hour	0	1	2	3	4
15. Sitting for 1 hour, like when in church, taxi or meetings	0	1	2	3	4
16. Fast walking on even ground	0	1	2	3	4
17. Fast walking/running on uneven ground	0	1	2	3	4
18. Making sharp turns while walking/running very fast	0	1	2	3	4
19. Standing up fast from squatting	0	1	2	3	4
20. Turning in bed	0	1	2	3	4
